# Strategies to increase adoption of animal vaccines by smallholder farmers with focus on neglected diseases and marginalized populations

**DOI:** 10.1371/journal.pntd.0006989

**Published:** 2019-02-07

**Authors:** Meritxell Donadeu, Nick Nwankpa, Bernadette Abela-Ridder, Baptiste Dungu

**Affiliations:** 1 Faculty of Veterinary and Agricultural Sciences, The University of Melbourne, Werribee, Victoria, Australia; 2 Initiative for Neglected Animal Diseases (INAND), Midrand, South Africa; 3 African Union-Pan African Veterinary Vaccine Centre (AU-PANVAC), Debre Zeit, Ethiopia; 4 Department of Control of Neglected Tropical Diseases, World Health Organization, Genève, Switzerland; 5 Independent Consultant, Edinburgh, United Kingdom; Wistar Institute, UNITED STATES

## Abstract

**Background:**

Most smallholder farmers (SHFs) and marginalized populations (MPs) in Africa, Asia, and Latin America depend on livestock for their livelihoods. However, significant numbers of these animals do not achieve their potential, die due to disease, or transmit zoonotic diseases. Existing vaccines could prevent and control some of these diseases, but frequently the vaccines do not reach SHFs, especially MPs, making it necessary for specific vaccine adoption strategies.

**Principal findings:**

Several strategies that have the potential to increase the adoption of animal vaccines by SHFs and MPs have been identified depending on the type of vaccines involved. The strategies differed depending on whether the vaccines were aimed at diseases that cause economic losses, government-controlled diseases, or neglected diseases. The adoption of vaccines for neglected diseases presents a major challenge, because they are mostly for zoonotic diseases that produce few or no clinical signs in the animals, making it more difficult for the farmers to appreciate the value of the vaccines.

Strategies can be aimed at increasing the availability of quality vaccines, so that they are produced in sufficient quantity, or aimed at increasing access and demand by SHFs and/or MPs. Some of the strategies to increase vaccine adoption might not provide a definite solution but might facilitate vaccine uptake by decreasing barriers. These strategies are varied and include technical considerations, policy components, involvement by the private sector (local and international), and innovation.

**Conclusions:**

Several strategies with the potential to reduce livestock morbidity and mortality, or prevent zoonoses in SHFs communities and MPs through vaccination, require the involvement of donors and international organisations to stimulate and facilitate sustainable adoption. This is especially the case for neglected zoonotic diseases. Support for national and regional vaccine manufacturers is also required, especially for vaccines against diseases of interest only in the developing world and public goods.

## Introduction

Many smallholder farmers (SHFs) in Africa, Asia, and Latin America depend on animals for their livelihoods. Livestock are critical to the livelihoods of poor livestock-keepers in the developing world, estimated to number between 600 and 900 million [1, 2]. Animals are critical because they provide food, income, status, and are the financial reserve for the family. Unfortunately, many of those animals die or do not achieve their productive potential, due to disease, affecting the income and livelihoods of millions of people. In addition, a number of diseases affecting livestock in poor communities are due to zoonotic pathogens, causing morbidity and mortality in the human population [3].

Some SHFs are members of communities that are particularly disadvantaged, either due to ethnic, religious, or cultural reasons; their lifestyle such as mobile pastoralists; because they are relatively few in number; or due to some other circumstances. These communities, which may be referred to as marginalized populations (MPs), are highly vulnerable, typically the poorest and those with the least access to health services. Poor communities living in rural remote areas and in marginal urban areas are prominent among MPs. Often these communities are overlooked by their own governments as well as by international agencies and donors, although they are becoming more visible due to the “Leave no one behind” agenda of the Sustainable Development Goals [4]. The dependence of MPs on their animals is particularly pronounced.

**Fig 1:**
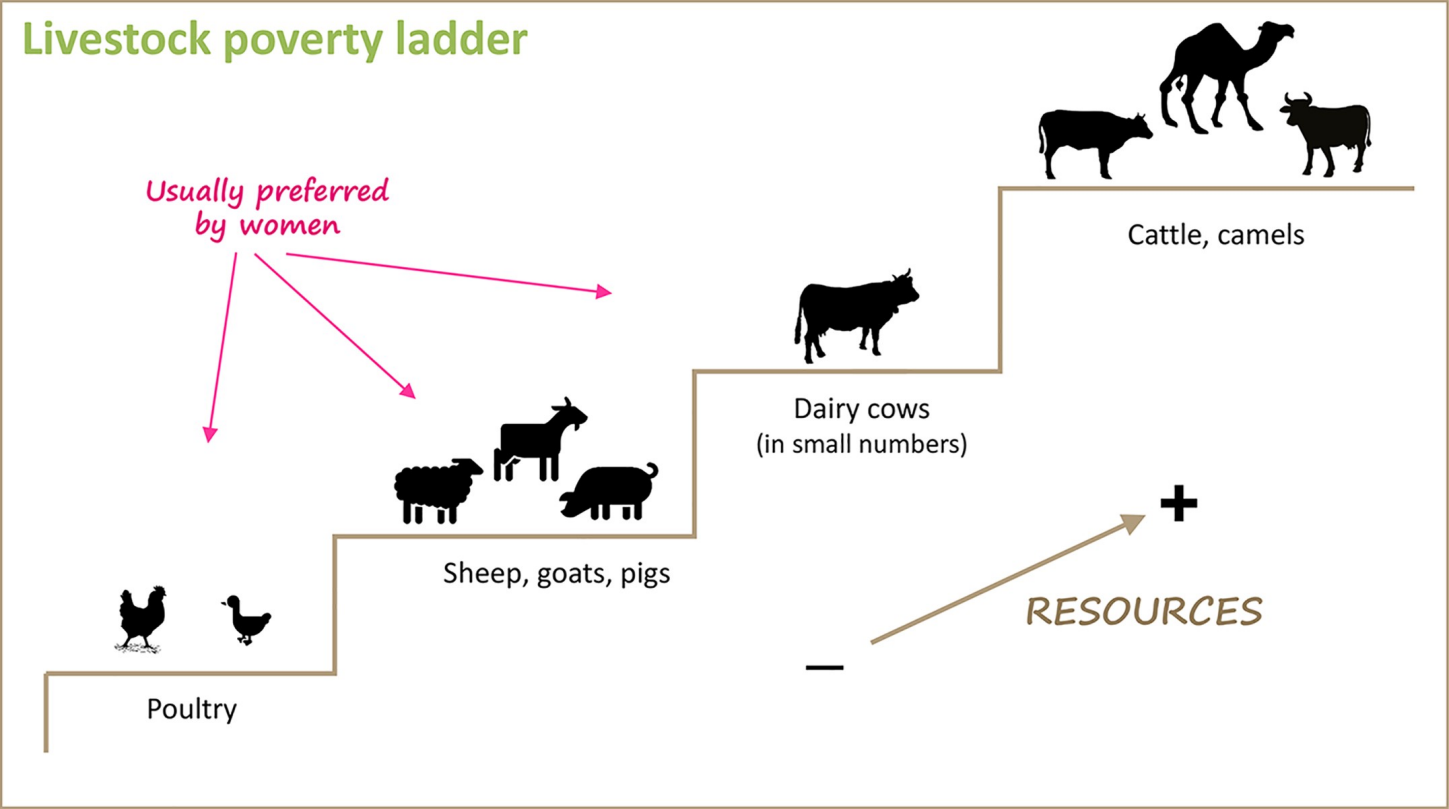
Livestock poverty ladder, species preference, and gender. Livestock species owned by the poor livestock keepers depend traditionally on their level of resources. Women usually prefer poultry, pigs, small ruminants and dairy cattle in small numbers, although there are exceptions. Some silhouettes obtained from: https://openclipart.org.

SHFs in developing countries can be considered to belong to different categories in terms of their wealth, on what is referred to here as the poverty ladder (Fig 1). The poorest SHFs at the bottom of the ladder tend to own only poultry. One step above, they tend to also own pigs or small ruminants (SRs; sheep or goats), depending on cultural and environmental factors. Less poor SHFs may own dairy cattle in small numbers, for example, in periurban areas, where families might have one or two milking cows. The SHFs with the most resources are the ones who own beef cattle, larger numbers of dairy cattle, or camels. Interventions aimed at improving the livelihood of SHFs that are directed at poultry, pigs, and SRs will benefit particularly those SHFs with fewer resources, including MPs.

Women comprise approximately 70% of the world’s poor, as well as the majority of poor livestock keepers [5]. Poultry, pigs, and SRs are the livestock species usually preferred by women because they can be more easily managed, and in many instances, the income generated goes directly to them [6–12]. Any intervention aimed at improving animal health in poultry, pigs, and SRs such as vaccination is likely to provide benefits particularly to women SHFs.

Vaccines are one of the most cost-effective and sometimes the only means to prevent disease in livestock populations, including zoonotic diseases. Commercial vaccines are available for prevention and control of many livestock diseases; however, these vaccines frequently do not reach, and thus are not often used by, SHFs. Here, we seek to identify strategies that can increase the adoption of animal vaccines by SHFs and MPs.

## Methodology

Reference was made specially to reports prepared by agencies such as the Food and Agriculture Organization (FAO) of the United Nations, the World Organisation for Animal Health (OIE), the World Health Organization (WHO), the authors’ previous work in the area [13], as well as literature reviews (PubMed) and internet searches. A simplified vaccine supply chain was considered, because the focus of this paper is restricted to vaccines that are currently produced commercially. The areas considered for the review were (1) manufacturing, (2) distribution, (3) uptake by commercial farmers, (4) uptake by SHFs and/or MPs. Other areas such as research, development, scaling-up, and registration were thus omitted. The uptake of vaccines by commercial farmers in developing countries was included, because some of the structures used by them could also benefit SHFs.

## General considerations

Animal diseases were categorized in three main groups to facilitate the analysis, as follows.

### Diseases that cause economic and/or production losses

Vaccines against these diseases are typically private goods (the term is used here to describe vaccines that farmers are willing to purchase). Within this group, there are two types of diseases:

- diseases of interest also in developed countries such as Newcastle disease (ND) and classical swine fever (CSF);
- diseases of interest only in developing countries such as contagious caprine pleuropneumonia (CCPP) or East Coast fever.

### Government controlled diseases

This usually includes notifiable and transboundary diseases such as foot and mouth disease (FMD) and Peste des petits ruminants (PPR). Vaccines for these diseases can be public (vaccines that farmers are not likely to purchase) or private goods. There is some variation between countries, and some diseases such as contagious bovine pleuropneumonia (CBPP) can fit in this category or the category above, depending on the country.

### Neglected diseases

In human health, some infectious diseases such as leprosy and Chagas disease are called neglected because they affect mainly the poor; they are associated with social determinants of health, for example, inadequate access to health services, education, safe water, and basic sanitation; and do not receive enough attention, even though most of these diseases are treatable and can be cured. As well as being important causes of morbidity and mortality, they contribute to significant stigma and discrimination in affected communities. These diseases are typically not prioritized by either governments or aid agencies, because they have other priorities (for example, Malaria, HIV/AIDS, tuberculosis). Nor are they prioritized by drug and vaccine developers and pharmaceutical companies due to their association with limited markets, likely low profitability, and poor chances of recovering the cost of developing new products.

Similarly, in animal health, there are also neglected animal diseases that affect mainly animals of poor and marginalized populations in low-resource settings, and for which there are not significant resources invested in trying to address them. Neglected animal diseases cause decreased productivity and animal mortality, and some are zoonotic, impacting the livelihoods of individuals and communities. Examples include *Taenia solium*, *Echinococcus granulosus*, and dog-transmitted rabies. Vaccines for these animal diseases are typically public goods.

Zoonotic diseases, especially endemic ones, are of special importance because they have a disproportionate impact on MPs [14, 15]. Zoonoses are represented in each group of diseases mentioned above.

Some diseases can be categorized into more than one group; for example, Rift Valley fever (RVF), which causes livestock production losses during outbreaks, is neglected during the inter-epizootic stages in many countries, even if it remains a significant zoonotic disease during this period.

### Weak links in the supply chain of existing animal vaccines

**Fig 2:**
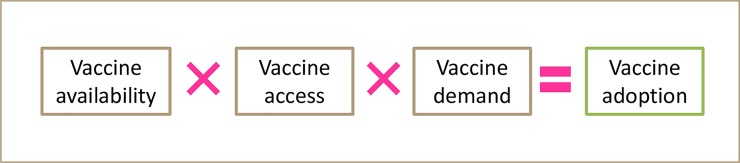
Vaccine adoption. Vaccine adoption is the result of vaccine availability, vaccine access, and vaccine demand [13]. Each component is required to achieve vaccine adoption. The figure assumes that the vaccine is safe and effective and that these are not limiting factors to vaccine adoption.

If livestock vaccines are to be adopted by SHFs and/or MPs, it is necessary that vaccines are available, accessible, and that there is a demand for them (Fig 2). Vaccines being available means that effective and safe vaccines exist, that they are produced at a large scale, and that they are purchasable on the market; therefore, availability refers to the product itself. Vaccines should also be accessible to SHFs, meaning that the farmers have access to them, for example, via local agro-shops or from vaccinators. The term accessible also incorporates the affordability aspect. Demand for vaccines refers to SHFs being aware of the existence of a vaccine and wanting to use it because they value the potential benefits of using the vaccine. It is the product of availability, access, and demand that determines the extent to which a vaccine is adopted [13] (Fig 2).

Supply chain links of existing animal vaccines for SHFs and/or MPs were analyzed according to the disease categories referred to above, to identify the areas in which vaccine adoption strategies should be targeted. Table 1 summarizes areas of weaknesses and the relative importance of the identified weaknesses.

**Table 1:** Weak links in the supply chain of existing animal vaccines according to the different disease categories. Increased level of weakness is represented by a larger number of +.

Vaccine supply chain Disease categories	Manufacturing	Distribution	Commercial use	SHFs/ MPs use
Cause economic losses also in developed countries	−^1^	−	−	++
Cause economic losses only in developing countries	+++^2^	++	++^3^	+++
Diseases controlled by governments	+^4^	?^5^	+^6^	+++
Neglected diseases	+++^7^ (likely to increase)	+++	N/A	+++++

^1^Not considered a weak link.

^2^Usually vaccines for this group of diseases are produced by local or regional manufacturers and not by large multinational companies. In most cases, there are few manufacturers and not sufficient production capacity to meet the demand.

^3^Commercial farmers usually will find a way to access the vaccines they need and may buy them directly from the manufacturer or the importer.

^4^These vaccines are produced by local manufacturers but can also be produced by large multinational companies and be imported. In many instances, they are procured through tenders.

^5^Distribution depends on government strategies.

^6^In some countries, farmers need to have a certificate of vaccination in order to sell the animals, so they are compelled to pay for the vaccine and will establish a supply mechanism.

^7^ There are only one, or a few manufacturers at global level, and for some products, continuity of production is at risk due to a lack of demand.

**Abbreviations:** MPs, marginalized populations; N/A, not applicable; SHFs, smallholder farmers.

## Potential strategies to increase vaccine adoption by SHFs and/or MPs

Strategies that could be implemented to increase vaccine adoption should not only consider the use by SHFs (access and demand) but also vaccine manufacturing strategies that will ensure adequate vaccine production (availability), because these are the main areas of weakness in the existing vaccine supply chains (Table 1). Vaccine availability is a barrier for adoption if the vaccine is not produced in sufficient quantity and good quality. Good quality vaccines are important to induce effective immunity. Table 2 summarizes different potential strategies that could secure vaccine production and increase vaccine use at SHFs and/or MPs level, according to the different disease categories.

**Table 2:** Potential strategies to secure vaccine production and increase vaccine use by SHFs and/or MPs according to the different disease categories. Strategies are generic and might not apply to all diseases in each group, or in all countries. The different strategies need to be considered within local, national, and regional contexts. Some strategies may only provide a partial solution. See text for details.

Group of diseases	Strategies at manufacturing level	Strategies to increase vaccine adoption by SHFs/MPs
Cause economic losses also in developed countries	Not applicable^1^	**Access:** 1. Creation of access points: creation of a sustainable supply via rural retailers and local authorized vaccinators. Might include access to capital by local retailers. 2. Establishment of community supply: vaccine supply through farmers associations and other community structures. 3. Institution of prize mechanisms: prize mechanisms for distribution (assuming there is capital available). Demand: 4. Increasing awareness of vaccine benefits and disease control programs: can include the development of vaccination programs. 5. Increased vaccine value: multivalent vaccines, integration of vaccines to technical support packages. Access and demand combined: 6. Technical considerations: small pack size and thermotolerance.
Cause economic losses only in developing countries	**Availability:** • Support local and regional vaccine manufacturers to ensure there is enough supply of quality vaccine.	**Access and demand:** As above, but when the private sector is involved, it will be mainly local; therefore, strategies that require access to capital might not work or might be limited in scale.
Diseases controlled by governments	**Availability:** • Support vaccine manufacturers to ensure there is enough supply of quality vaccine.	**Access and demand:** 1. Implementation of government policies that promote the use of vaccines. 2. Provide partially or fully subsidized vaccines only to SHFs/MPs and not to commercial farmers. 3. Ensure vaccination policies promote cooperation amongst farmers and do not interfere with other vaccination strategies. 4. Strengthening of the national veterinary services.
Neglected diseases	**Availability:** • Purchase guarantee (but this will not guarantee distribution or vaccine use). • Ensure demand for the vaccine.	**Access:** 1. Creation of vaccine and antigen banks or stockpiles (strategic reserves). Demand: 2. Transformation of public goods into private goods: combination vaccines, bundle products, expansion of label claims. 3. Development of disease control guidelines and large-scale demonstrations: to encourage governments to take ownership and leadership 4. Demonstration of the benefit of neglected zoonotic disease control programs. 5. Social participation and community engagement. Access and demand combined: 6. Integration with other animal health activities. 7. Integration with human health sector providers and other human health interventions. 8. Technical considerations: One-shot vaccines (very important to reach remote populations), thermotolerance, ease of application. 9. Institution of prize mechanisms: to stimulate technological platforms of interest.

^1^Not considered to be a weakness in the vaccine supply chain.

**Abbreviations:** MPs, marginalized populations; SHFs, smallholder farmers.

### Strategies to improve adoption of vaccines for diseases associated with economic and/or production losses

It is easier for farmers to appreciate the value of a vaccine if it prevents a disease that causes a clear economic or production loss, especially if the disease causes high mortality. Cost of the vaccine does not tend to be an issue if it is within reason from the SHF’s perspective. The options to increase adoption of vaccines in these circumstances are the following.

#### Strategies to increase vaccine access

**1. Creation of a supply chain and access points.** In many cases, vaccines do not reach the rural retailers and/or farmers due to lack of access. Creating a sustainable supply chain linking wholesalers to rural retailers, and rural retailers with animal vaccinators (veterinarians, community animal health workers [CAHWs], or authorized technicians), can overcome this issue. Achieving this may require leadership by a private company or a nongovernmental organization (NGO) to stimulate the process. Some financial mechanisms such as providing access to capital to the small retailers (for example, to secure a cold chain facility) might also be required.

This strategy has been used for distribution of ND vaccine [13], and is the basis for operation by SIDAI in Kenya, a network of at least 150 franchises owned and managed by qualified livestock professionals.

**2. Establishment of community supply.** Farmers’ associations may purchase veterinary medicines and kits to facilitate supply to local SHFs (with adequate veterinarian supervision). Because they buy in bulk, price discounts might be possible to incentivise vaccination at the appropriate time. This strategy has been used in pastoralist communities in the north of Argentina.

**3. Institution of prize mechanisms.** Prize mechanisms offered by philanthropic organizations and others to stimulate sustainable supply and distribution systems can be considered but that would assume that capital is available to support the initiative, which may often not be the case.

#### Strategies to increase vaccine demand

**4. Increase awareness of the benefits of vaccines and disease control programs.** Many SHFs are not aware of the benefits of the vaccines, and an increased awareness can stimulate demand. This can be achieved through education by a variety of mechanisms, including radio campaigns, product demonstrations, etc. Awareness through the development of appropriate vaccination programs, tailored to the needs of SHFs, have proved valuable in encouraging vaccine usage. For example, in South Africa through Afrivet Training Services, farmers have been trained on the value of vaccination and the value of following an appropriate vaccination program.

**5. Increase vaccine value to make them more attractive.** This can be done through the use of multivalent vaccines or integrating vaccines to SHFs’ technical support and extension packages.

#### Strategies to increase both access and demand

**6. Technical considerations (use of thermotolerant vaccines and vaccines packaged in smaller number of doses).** Vaccines in packages containing a small number of doses are preferable to SHFs; they are more cost effective and prevent wastage. Thermotolerance of the vaccines will ensure the quality of the product at the time of vaccination, especially in areas where maintaining the cold chain is a challenge.

Some of the strategies mentioned can be supported by paraveterinary professionals, especially CAHWs. CAHWs play an important role as they facilitate the vaccine reaching the target population. Regrettably, CAHWs often lack legal support, and they face several challenges [16]. It is important for the sustainability of CAHW that they make enough money on a continuous basis from their work. Some of the strategies that can help achieve this—such as a correct pricing structure to ensure suitable margins, providing solution packages and provision of the right vaccine accessories—have been described elsewhere [13]. Empirical data indicate that CAHWs are desirable because they offer the needed care and attention that farmers want, are located close to the farmers, have lower transaction costs, and are likely to be trusted by the farmers [17]. This may not always be the case; for example, a survey in Ghana demonstrated that government paravets were preferred to CAHWs because limited training of the CAHWs led to them having higher costs and poor performance [18]. Tools to evaluate the performance of paraveterinary professionals are included in the OIE Tool for the Evaluation of Performance of Veterinary Services (OIE PVS Tool). They can be supplemented taking into consideration stakeholders’ expectations and perceptions as well as national animal health objectives using participatory tools such as those used in Cambodia [19] and Uganda [20].

The strategies may be effective with SHFs but probably not with the most MPs because they would be unlikely to afford any vaccination costs, making the support of government and philanthropic agencies necessary.

### Strategies to increase vaccine adoption for the group of diseases that produce economic losses only in developing countries

For diseases causing economic losses only in developing countries, the manufacturing (and consequent availability) of vaccines may be a bottleneck to vaccine supply. Often these vaccines are produced by local or regional manufacturers and not by multinational companies. Local or regional manufacturers can be private, but in many countries, especially in Africa, they are government owned. The challenges they face arise from a low production capacity (in some cases, inadequate infrastructure, technical capacity, or technical knowledge) and/or a lack of demand [13]. Many of them also produce vaccines against diseases that cause economic losses in developed and developing countries, because these vaccines are most often profitable. However, a major problem that is growing is that the revenue of local manufacturers from vaccines that also cause economic losses in developed countries is increasingly being eroded by competition from multinational companies that are extending their range of activities to these areas, targeting the most profitable vaccines. Therefore, the local laboratories are losing earnings from the profitable vaccines that contribute substantially to cover their production fixed costs and are left with the much needed but less profitable vaccines (including the vaccines for diseases of economic importance only in developing countries and public-good vaccines). Strategies to support local manufacturers should be encouraged, especially as already indicated; they are the major manufacturers of vaccines against many public-good diseases and diseases affecting exclusively developing countries.

To increase the use of vaccines against infections that only cause losses in developing countries, strategies similar to the ones already described in the section above can be used. However, participation of the private sector will likely be limited only to the local private sector (multinational companies do not tend to produce vaccines for these diseases). In many instances, only the government-owned vaccine manufacturing facilities will be involved. These actors are more capital constrained than the multinational companies; therefore, strategies such as awareness campaigns and creation of supply chains may be difficult to implement without access to financial support.

### Strategies to improve adoption of vaccines for government-controlled diseases

For the diseases that are government controlled, vaccine usage by SHFs is determined by government policy. Some policies such as the requirement of vaccination certificates to move, sell, or slaughter animals encourage the use of the vaccines.

Although government policies may dictate vaccination for certain diseases, constraints may exist when governments provide vaccines at no cost to farmers, especially in situations in which the government does not have resources to provide vaccines in sufficient quantity and in a timely manner. In some other countries, farmers are compelled to pay for vaccines that are government controlled, such as the FMD vaccine. Potential strategies that require large commercial farmers to pay for the vaccine, whereas SHFs receive it at partially or fully subsidised prices, might be an alternative solution to government paying for all farmers or all farmers paying.

It is important that there are government policies to support and encourage livestock vaccination. Onono and colleagues, 2017 [21], undertook an analysis of policy on the delivery of the CBPP vaccine and suggested the adoption of contractual agreements between the public and private sectors to support the vaccination of susceptible herds. The authors suggest that this policy would increase vaccination coverage of susceptible cattle herds. Policies should also encourage cooperation amongst farmers, and they should not interfere with other strategies.

Initiatives to strengthen the national veterinary services, such as those promoted by the OIE through the performance of veterinary services (PVS) system [22], should be recommended, because robust veterinary services will be in a better position to develop and implement suitable adapted policies, including vaccination policies.

### Strategies to improve adoption of vaccines for neglected diseases

Neglected diseases, including neglected zoonotic diseases (NZDs), are neglected because they affect mainly the poor, they are not subject to compulsory reporting in most countries, and they are not perceived as major public health burdens as compared to HIV/AIDS, tuberculosis, and malaria. Most of them do not lead to epidemiological emergencies and consequently, attract little attention from the media, donors, and the public sector. The private sector does not consider this group as a lucrative target [23]. Some of the key examples of animal vaccines in this group are the vaccines for porcine cysticercosis (used to stop the transmission of *T*. *solium*, a parasite that causes epilepsy in humans), and the sheep vaccine for *E*. *granulosus* (which produces hydatid disease and cystic echinococcosis in people). For both diseases, there is little incentive for the farmers to vaccinate their animals, because the animals show no sign of the disease and do not have additional economic value after vaccination. Another example is RVF; during an epizootic outbreak, animals will be sick and SHFs might be interested in the RVF vaccine, but during the long inter-epizootic outbreaks, there is no incentive or motivation for farmers to use the vaccine.

The case for the control of zoonoses—including NZDs—using the One Health approach as an equitable and effective method to achieve improved health in low and middle-income countries has been presented by Cleaveland and colleagues [24]. They highlight that although treatments are available for several endemic zoonoses, it is the disadvantaged and the poor who are “left behind.” Although a wide range of potential interventions may be considered that could improve the use of vaccination against NZDs, particular opportunities exist in relation to livestock vaccination strategies in Africa.

There is little or no commercial interest for the manufacturing of NZDs livestock vaccines. A similar situation is encountered with vaccination against many of the neglected diseases of humans; with the exception of some viral neglected tropical diseases, there has been minimal industry interest, leaving them to a handful of nonprofit product development partnerships [25].

Strategies to incentivize the manufacturers to produce livestock vaccines for NZDs, such as a prize guarantee or purchase guarantee offered by philanthropic organizations and others, will not be sustainable in the medium to long term and will not ensure that the vaccine reaches SHFs and/or MPs unless other mechanisms for deployment are in place. The main incentive for vaccine manufacturers is their corporate social responsibility, because profit margins are small, and demand is erratic and extremely limited. Ensuring demand is probably the main strategy to incentivize the manufacturers to continue producing the vaccine; this is critical, especially for vaccines such as porcine cysticercosis in which there is only one manufacturer worldwide and there is a substantial risk that the vaccine could be discontinued if there is a lack of demand.

Options to increase or facilitate the demand for livestock NZDs vaccines by SHFs and/or MPs can include the following.

#### Strategies to increase vaccine access

**1. Creation of vaccine and antigen banks or stockpiles.** The vaccine bank for the dog rabies vaccine created by OIE has proven to be very successful in breaking the high-price–low-demand cycle and generating a demand and supply cycle that can be reliably quantified [26]. By creating the vaccine bank and buying vaccine in large quantities, lower prices have been achieved. The funds of the OIE Rabies Vaccine Bank cover vaccine production expenses and transportation costs to the beneficiary countries so that countries can focus their resources in other necessary areas, such as raising awareness of the disease, managing stray dog populations, and increasing access to treatment for humans [27]. From inception to October 2017, over 19.1 million doses of rabies vaccines have been delivered or ordered for eligible countries in Africa and Asia [28].

This stockpile strategy could also work with other NZDs such as RVF. RVF is a cyclic disease that manifests in major outbreaks with long interepidemic periods of up to 15 years, depending on various risk factors [29, 30]. Some countries conduct annual or biannual vaccinations, others vaccinate following warnings of imminent RVF outbreaks, and yet some countries with sporadic outbreaks do not have control strategies [31]. Due to its irregular and cyclical nature, SHFs do not use the vaccine routinely and when the need arises in the face of an outbreak, there is usually not enough vaccines available. A rolling stock of vaccines managed at a national or regional level could constitute a solution especially in countries without active RVF circulation [32, 33]. There have been discussions to set up an antigen stockpile at the OIE or the Southern African Development Community (SADC), but nothing has materialized so far.

#### Strategies to increase vaccine demand

**2. Transforming public goods into private goods.** This can be done by combining vaccines for NZDs that are considered public goods with vaccines or other products that are of interest to farmers. For example, the hydatidosis vaccine for sheep could be combined with the clostridial vaccine (in some places, SHFs are willing to pay for the clostridial vaccine). RVF vaccines could be combined with vaccines for other ruminant diseases of interest for SHFs. In Latin America and South Asia, the porcine cysticercosis vaccine could be combined with CSF vaccines. Creation of these new combined vaccines would require technical issues to be evaluated and optimized—for example, antigen or adjuvant compatibility, different timing of vaccination schedules, or the number of vaccinations needed to trigger immunity.

These options to transform public goods into private goods are limited, because these diseases usually affect MPs that generally will not be able to afford any animal vaccine at all.

**3. Developing disease control guidelines and implementing large-scale demonstrations.** The implementation of NZDs control programs is usually based on government (national or sub-national) programs, supported by the governments themselves and by NGOs or philanthropic organizations. One of the key constraints for governments is the lack of knowledge and guidelines for the implementation of control programs for some NZDs using animal vaccines. Although this knowledge is available for some diseases, such as dog-mediated rabies, it is not so clear for other diseases, such as cysticercosis and hydatid disease. The technical information is available at an individual level, but it is not available for control programs at population level; for example, which animal categories should be vaccinated, with which frequency, and at what period of the year? what should be the target coverage for vaccinations? This lack of knowledge generates reluctance in governments to set up control programs.

Generation of this kind of knowledge is one of the key challenges. Governments usually do not have the technical expertise or resources to generate this information, and researchers and practitioners find it difficult to get the necessary support to conduct such programs. Donors prefer to support more basic science projects, and many believe that when the vaccine is available, it is up to the farmers and governments to use it. Support, for example, has been obtained to do field validations of the cysticercosis vaccine but very little to conduct large-scale control programs that are simple enough for governments to replicate or scale up.

These control program demonstrations would be extremely useful for governments to learn key lessons, identify the main challenges and solutions, and obtain data that will facilitate the better implementation and scale up to other parts of the country. Such large-scale demonstrations have been implemented for rabies in Tanzania [34]; even if rabies was not eliminated, there were major declines in dog bites and suspected rabies cases, with a concomitant fall in the demand for postexposure prophylaxis (PEP). Lessons were learned on logistics, coordination, management, and technical issues that are of great value for future programs.

**4. Demonstration of the value and benefits of NZDs control programs.** Many governments and donors are only keen to support control programs when there is enough data on the effectiveness of such control programs. An important challenge is the lack of sufficient data to generate evidence, particularly from programs that incorporate new vaccines. Data from small-scale vaccine trials are usually not sufficient to provide a clear cost/benefit analysis, and there are insufficient resources to generate this data, which creates a vicious cycle. Estimates, data generated in other countries, and prediction models can provide some useful information but have important limitations.

Several tools have been developed to assist in this area. A One Health Framework has been created to facilitate the estimation of the economic costs of zoonotic diseases [35]. The most frequently cited measure to assess an intervention is the cost-effectiveness ratio based on cost per disability-adjusted life year (DALY) averted. An example is the assessment of the human health benefits from livestock brucellosis vaccination in Mongolia [36]. Other measures have also been used, such as the separable cost method, the cost-benefit analysis, and more recently, the zDALYs, (a modified DALY for zoonotic diseases) which have been developed to better evaluate the burden of zoonotic diseases [37]. The strengths and weaknesses of each have been evaluated [38]. The zDALY includes animal loss equivalents (ALEs), which is the monetary value of animal health losses divided by the gross national income (GNI) per person, as well as the DALY components. These measures can be very useful, but caution needs to be applied when using these parameters in relation to diseases with focal distribution, or in MPs that are affected in a disproportionate way, to avoid a “population dilution” factor. For example, evaluating the cost effectiveness of a *T*. *solium* intervention in the Dalit communities of India and Nepal might be diluted by the relatively large population of other castes at less risk of cysticercosis in those countries. The pig-keeping Dalit communities are an example of a community that is disproportionately affected; in 2007, it was reported that 20% of the Dalits didn’t have access to safe drinking water, only 9.8% had access to sanitation (compared to 26% for other castes), and 30%–40% of the villages surveyed reported that public health workers refused to visit Dalit villages [39]. Therefore, using the national and/or regional statistics might not truly reflect the impact in these MPs.

**5. Social participation and community engagement.** Creating awareness in the community and in the wider group of stakeholders of the issues and the solutions involving vaccines is a potential way to increase demand. This awareness might be created by governments (assuming they have the resources and it is aligned with their priorities), but might also be created by NGOs and philanthropic organizations.

#### Strategies to increase both vaccine access and demand

**6. Integration of vaccinations for NZDs with other animal health activities.** Logistics are a key constraint for vaccine adoption, and efforts towards their simplification and cost reduction can prove very valuable. Vaccinations for NZDs might be combined with other animal health activities for which logistics and other costs are already covered. For example, the vaccine for RVF or the vaccine for hydatid vaccine could be combined with vaccines for the current global strategy for the control and eradication of PPR [40]. Technical challenges similar to the ones faced with multivalent vaccines would need to be overcome, such as the time of vaccination or the number of doses required to trigger protective immunity.

**7. Integration of vaccinations with the activities of human health sector providers and other human health interventions.** Community health workers (CHWs) in the human sector have been identified as one strategy to address the shortage of health workers, particularly in low-income countries. The roles and activities of the CHWs are enormously diverse within and across countries and within programs. They can make a valuable contribution to the community if appropriately trained and supported [41]. It is possible to integrate the work of CHWs to interventions in the control of human and animal diseases, especially NZDs. Integration might range from only sharing logistics to sharing staff. Joint human and animal vaccination campaigns have been conducted in pastoralist settings (see “The case of nomadic pastoralist communities” below), and recent studies about community perceptions on integrating animal vaccination and public health workers in Uganda showed that participants believed that services could work together, although some caveats were expressed [42]. Delivery of this strategy would be facilitated by strengthening national veterinary services, as is being promoted the OIE [22], as well as strengthening human health services, which is being promoted by WHO [43].

**8. Technical considerations.** The use of thermotolerant vaccines and vaccines less reliant on cold chain are an added advantage, but when dealing with MPs, more emphasis should be placed on vaccines that can trigger protective immunity following one immunization. Marginalized communities are often in remote areas, and logistics, including the gathering of roaming and/or pasturing animals, present enormous limitations, more so than vaccine cost or the cold chain. One-shot vaccines could also facilitate the integration of NZD vaccinations with other animal health activities.

While thermotolerant vaccines become available, it is important to increase the capacity of the cold chain to keep vaccines at optimal temperatures during longer periods of time and ensure vaccine quality. For example, better insulation boxes with novel materials could simplify logistics.

**9. Prize mechanisms.** Prize mechanisms, in which a monetary incentive is given when reaching certain milestones, are currently being used to improve the vaccine for *Brucella melitensis* under an AgResults multilateral initiative [44] to stimulate the participation of the private sector. This mechanism is likely to be limited to vaccines that are of interest to the private sector, and vaccines would need to have application beyond SHFs and/or MPs. A prize mechanism is not likely to be effective for vaccines that relate only to NZDs. Prize mechanisms could also be valuable to stimulate technological platforms that can help overcoming technical challenges, such as one-shot vaccines or thermotolerance, because these technologies could also be applied to products of interest to the private companies.

### The case of nomadic pastoralist communities

The mobility and dispersion of nomadic pastoralist communities presents major operational and resource challenges to the provision of basic veterinary services. CAHWs have proven successful in pastoralist areas [45], but they have limited coverage. Mobile veterinary services have, and are being used, although they are not always successful [46]. A recent review of institutional arrangements to provide animal health services in Kenya and Uganda calls for the establishment of public veterinary systems for the provision of veterinary services in pastoral areas [47]. Challenges involved in delivering health services in these communities are applicable to humans and animals, so under One Health approaches, the strategy described in seven above of integrating human and animal health services, would be of particular interest. In Chad, joint campaigns in nomadic communities demonstrated the feasibility of combining vaccination programs for people and their livestock, extending the capacity of existing mobile veterinary teams for simultaneous vaccination of people and animals [48]. These joint campaigns reduced the total costs, and more children and women were vaccinated per day during joint vaccination rounds than during vaccinations of people only. Increased coverage of vaccination of children was also observed in southern Sudan when undertaken together with vaccination of cattle for rinderpest [46], and more recently in Nigeria [49] with joint vaccination of people and animals.

### The role of innovation and the private sector in vaccine adoption by SHFs and/or MPs

Innovation will play a vital role in increasing the adoption of animal vaccines by SHFs and/or MPs. However, to date, the major emphasis of schemes to increase innovation have been made at the top of the vaccine chain, that is, in research and development (R&D). It is important to include innovation at all stages of the vaccine chain, including deployment. Often it is not the existence of an effective vaccine per se that limits the usefulness and value of a vaccine, especially in MPs. Other factors that affect availability, access, and demand often play a vital role, and there is a great need for investment in innovation at all levels of the vaccine chain.

It is important to differentiate between the international and local private sectors, because they have different incentives and their main markets are different. The private sector has an important role to play; it is the provider of animal vaccines of commercial interest. In some cases, vaccines for NZDs have been developed by the private sector at its own expense or with considerable internal inputs. These few manufacturers that are prepared to invest in NZDs vaccines should be encouraged and supported.

The efforts of the private sector have been focused mainly on vaccine R&D and vaccine production and have rarely incorporated distribution and promotion of products for SHFs and/or MPs. The international private sector should be encouraged to participate at all levels of the vaccine chain; however, promotional activities with the private sector should not be undertaken at the expense of the local sector, especially when it is fragile and incipient. In many cases, the local private sector exists but lacks capital or business knowledge to develop. Funding mechanisms through philanthropic agencies should not promote competition, but they should rather consider the long-term impact on the local sector. Currently, there are several donor initiatives supporting international companies to expand in Africa; the impact of these initiatives on the local private sector and in the availability of public goods in the long term is to be seen, but concerns have been raised.

## Conclusions

The adoption of animal vaccines by SHFs and especially MPs to reduce livestock morbidity and mortality, and to control and prevent NZDs, could be increased by diverse strategies. These strategies vary depending on the disease, the local context, and the opportunities available. Some of the strategies described will facilitate the adoption of the vaccines but will not necessarily ensure or guarantee the vaccine use; they might be, however, extremely useful at removing some of the key barriers.

Strategies aimed at increasing the adoption of vaccines of commercial interest might be supported by the private sector and the SHFs themselves but might not work in MPs as these populations will be unlikely to afford any vaccine. Supporting local and regional manufacturers is of critical importance, especially for vaccines of diseases that are only present in developing countries and for many NZDs in which the global demand is low. Many of the strategies aimed at increasing adoption of vaccines for NZDs require international and philanthropic support, for example, to stimulate and support governments to establish disease control programs.

**Key learning points**

1. Existing vaccines that can prevent livestock diseases affecting animals owned by SHFs and MPs are often not adopted, affecting the animals and the livelihood of people who depend on their animals.
2. New strategies are required to increase adoption of animal vaccines by SHFs and MPs; they should consider the types of diseases at which the vaccines are aimed and differentiate between vaccines against diseases that cause economic losses, vaccines for government-controlled diseases, and vaccines for neglected diseases.
3. Strategies to increase vaccine adoption need to be varied and should include technical considerations, policy, involvement by the private sector (local and international), social participation, and innovation.
4. Neglected diseases, especially NZDs, have a high impact on MPs, and there is little incentive for farmers to vaccinate their animals, because their livestock may not show clinical signs of the disease. The global animal health industry does not consider vaccines against this group of diseases as a profitable target. These vaccines are usually public goods used in disease control programs; therefore, the role of governments and philanthropic organizations in their adoption is essential.
5. Supporting local and regional manufacturers is very important, especially for vaccines against diseases that are only present in developing countries and for many NZDs in which the global demand is low.

**Key papers in the field**

1. World Health Organization. The control of Neglected Zoonotic Diseases–a route to poverty alleviation. Geneva, 2005.
2. Shaw APM, Rushton J, Roth F, Torgerson PR. DALYs, dollars and dogs: how best to analyse the economics of controlling zoonoses. Rev Sci Tech. 2017;36(1):147–61.
3. Donadeu M, Dungu B. Market Development & Adoption: Lessons learned and observations during Phase 1 of Protecting Livestock, Saving Human Life (PLSHL-1) programme. 2013. Available from: http://vetvac.org/galvmed/docRep/docs/20_GALVmed_MDA_Lessons_Learned_PLSLHL_1__2013_EXTERNAL.pdf. [cited 2018 February 1].
4. Ilukor J. Improving the delivery of veterinary services in Africa: insights from the empirical application of transaction costs theory in Uganda and Kenya. Rev Sci Tech. 2017;36(1):279–89
