# Correction: Strategies to increase adoption of animal vaccines by smallholder farmers with focus on neglected diseases and marginalized populations

**DOI:** 10.1371/journal.pntd.0007279

**Published:** 2019-03-18

**Authors:** The PLOS Neglected Tropical Diseases Staff

There is an error in Table 2. In the far-right column, the phrases “Access,” “Demand,” “Access and Demand,” and “Access and Demand combined” should all be in bold. The publisher apologizes for this error. Please see the correct Table 2 here.

**Table 2:** Potential strategies to secure vaccine production and increase vaccine use by SHFs and/or MPs according to the different disease categories.

Group of diseases	Strategies at manufacturing level	Strategies to increase vaccine adoption by SHFs/MPs
Cause economic losses also in developed countries	Not applicable^1^	**Access:** 1. Creation of access points: creation of a sustainable supply via rural retailers and local authorised vaccinators. Might include access to capital by local retailers. 2. Establishment of community supply: vaccine supply through farmers associations and other community structures. 3. Institution of prize mechanisms: prize mechanisms for distribution (assuming there is capital available). **Demand:** 4. Increasing awareness of vaccine benefits and disease control programs: can include the development of vaccination programs. 5. Increased vaccine value: multivalent vaccines, integration of vaccines to technical support packages **Access and Demand combined:** 6. Technical considerations: small pack size and thermotolerance.
Cause economic losses only in developing countries	**Availability:** • Support local and regional vaccine manufacturers to ensure there is enough supply of quality vaccine.	**Access and Demand:** As above, but when the private sector is involved, it will be mainly local; therefore, strategies that require access to capital might not work or might be limited in scale.
Diseases controlled by governments	**Availability:** • Support vaccine manufacturers to ensure there is enough supply of quality vaccine.	**Access and Demand:** 1. Implementation of government policies that promote the use of vaccines. 2. Provide partially or fully subsidized vaccines only to SHFs/MPs and not to commercial farmers. 3 Ensure vaccination policies promote cooperation amongst farmers and do not interfere with other vaccination strategies. 4. Strengthening of the national veterinary services.
Neglected diseases	**Availability:** • Purchase guarantee (but this will not guarantee distribution or vaccine use). • Ensure demand for the vaccine.	**Access:** 1. Creation of vaccine and antigen banks or stockpiles (strategic reserves). **Demand:** 2. Transformation of public goods into private goods: combination vaccines, bundle products, expansion of label claims. 3. Development of disease control guidelines and large-scale demonstrations: to encourage governments to take ownership and leadership. 4. Demonstration of the benefit of neglected zoonotic disease control programs. 5. Social participation and community engagement. **Access and Demand combined:** 6. Integration with other animal health activities. 7. Integration with human health sector providers and other human health interventions. 8. Technical considerations: One-shot vaccines (very important to reach remote populations), thermotolerance, ease of application. 9. Institution of prize mechanisms: to stimulate technological platforms of interest.

^1^ Not considered to be a weakness in the vaccine supply chain.

**Abbreviations:** MPs, marginalized populations; SHFs, smallholder farmers.
